# CompaGB: An open framework for genome browsers comparison

**DOI:** 10.1186/1756-0500-4-133

**Published:** 2011-05-04

**Authors:** Thomas Lacroix, Valentin Loux, Annie Gendrault, Jean-François Gibrat, Hélène Chiapello

**Affiliations:** 1 INRA UR1077, Unité Mathématique, Informatique & Génome, Jouy-en-Josas, France

## Abstract

**Background:**

Tools to visualize and explore genomes hold a central place in genomics and the diversity of genome browsers has increased dramatically over the last few years. It often turns out to be a daunting task to compare and choose a well-adapted genome browser, as multidisciplinary knowledge is required to carry out this task and the number of tools, functionalities and features are overwhelming.

**Findings:**

To assist in this task, we propose a community-based framework based on two cornerstones: (i) the implementation of industry promoted software qualification method (QSOS) adapted for genome browser evaluations, and (ii) a web resource providing numerous facilities either for visualizing comparisons or performing new evaluations. We formulated 60 criteria specifically for genome browsers, and incorporated another 65 directly from QSOS's generic section. Those criteria aim to answer versatile needs, ranging from a biologist whose interest primarily lies into user-friendly and informative functionalities, a bioinformatician who wants to integrate the genome browser into a wider framework, or a computer scientist who might choose a software according to more technical features. We developed a dedicated web application to enrich the existing QSOS functionalities (weighting of criteria, user profile) with features of interest to a community-based framework: easy management of evolving data, user comments...

**Conclusions:**

The framework is available at http://genome.jouy.inra.fr/CompaGB. It is open to anyone who wishes to participate in the evaluations. It helps the scientific community to (1) choose a genome browser that would better fit their particular project, (2) visualize features comparatively with easily accessible formats, such as tables or radar plots and (3) perform their own evaluation against the defined criteria. To illustrate the CompaGB functionalities, we have evaluated seven genome browsers according to the implemented methodology. A summary of the features of the compared genome browsers is presented and discussed.

## Introduction

The diversity of tools available for visualizing and browsing genomic data has increased dramatically over the last years: Bluejay [1], GenoMap [2], GenomeComp [3], GenomeViz [4], DiProGB [5] to cite but a few. Projects aiming at supplanting or complementing current genomes browsers (GBs) are blooming as well. Although these different GBs provide the basic functionalities for browsing annotations on a genomic scaffold, their philosophy, functionality, interoperability and implementation are often unique or dedicated to a particular scientific context. Indeed, most of them have been developed to fulfil the specific needs of a particular lab and as a result, the current "landscape" of GBs is fragmented [6]. Such disparities make direct comparisons difficult and external labs interested in integrating an existing GB to their own projects often ends up making their choice based on arbitrary decisions. Attempts to categorize and compare GBs features have already been made, thus highlighting the need for guidance and clarity in this matter [7-9]. Though very informative, we believe those state of the art reviews lack the sustainability, flexibility and modularity of a dedicated framework. In this paper we propose a traceable methodology based on generic and specific criteria and a web application to perform GBs evaluations and comparisons. To the best of our knowledge, this is the first effort to compare GBs features using an open source standard methodology and to set up a community resource centred on this type of information. To illustrate the use of the proposed methodology, we provide hereafter a short description of the comparison of seven GBs.

## Methods

The methodology we developed is inspired by the Qualification and Selection of Open Source software (QSOS) method [10], which is designed to qualify, select and compare free and open source software in an objective, traceable and argued way. QSOS provides tools for defining a list of criteria, evaluating softwares, and a web server to visualize and compare the evaluations as tables or radar graphs. It also offers the possibility of weighting the criteria to fit a user specific context. The GB evaluation procedure we designed includes the 65 criteria provided in the generic section of QSOS version 1.6, which was designed for evaluating the potential and ease of integration of the software in a project.

Because QSOS is based on XML files, it has some constraints: the management of the data is tedious, user functionalities are limited (for example user comments are not possible) and data integration is difficult. This choice of implementation complicates the application of such a methodology, which is based on iteratively refining the loop: definition, evaluation, qualification and selection. To overcome these limitations, we designed a relational database (using PostgreSQL) and developed our own version of the web application with Ajax technologies (using GWT).

## Implementation

To cover the range GB features, we built a list of 60 criteria tailored for GBs specificities. Three levels of scoring (full, limited/medium and poor) were defined specifically for each criterion to discriminate as objectively as possible between the different capabilities. Some criteria are for information purposes only (no score), e.g. the type of application. The new criteria were classified in four sections: (1) technical features, including criteria such as the type of application, ease of installation, performance, supported platforms, interoperability, security; (2) data content and connectivity, including criteria such as the possibility to display private data, supported formats, connectivity with databases or web services, export features; (3) graphical user interface (GUI), deals with criteria such as visualization techniques, richness of widgets, degree of customization, ease of navigation, comparative genomic features; (4) annotation editing and creation: includes criteria about the possibility of collaborative annotation, function assignment using a controlled ontology and assessment of the quality of the annotation. All these criteria are fully described and documented in the CompaGB website.

We then designed the CompaGB database and web interface to allow consistent management of criteria, evaluations, user accounts, comments and suggestions. The framework also allows for multiple concurrent evaluations of the same software (see discussion in conclusion). The CompaGB web site offers advanced functionalities for visualizing GB comparisons as a radar graph or in a table format. Just like in the QSOS methodology, the user has the possibility to weight the criteria (i.e.: unimportant, average importance, essential) to reflect a particular context of utilisation and therefore to modulate the results accordingly. Three predefined profiles (biologist, computational biologist, computer scientist) are proposed as an example. Additional functionalities were also implemented in the CompaGB web site to provide all the tools needed for registered users to perform, manage and integrate new evaluations.

## Testing of the methodology: analysis of seven evaluated GBs

To test the developed methodology, four different evaluators have evaluated a total of seven different GBs: MuGeN (version 20060919) [11], GBrowse (version 2.15) [12], UCSC genome browser (version of January 2011) [13], Ensembl (version 54) [14], Artemis (version 13)/ACT (version 10) [15], JBrowse (version 1) [16] and Dalliance (version 0.5) [17]. GBs were chosen such as to cover a broad variety of functionalities: from simple and easily accessible software developed by a local team (MuGeN) to a representative selection of the most popular and sophisticated GBs that are used by a large community of biologists around the world (GBrowse, Ensembl). We also included two recent tool from the last generation of GBs that were developed with Ajax technologies: JBrowse and Dalliance.

**Figure 1:**
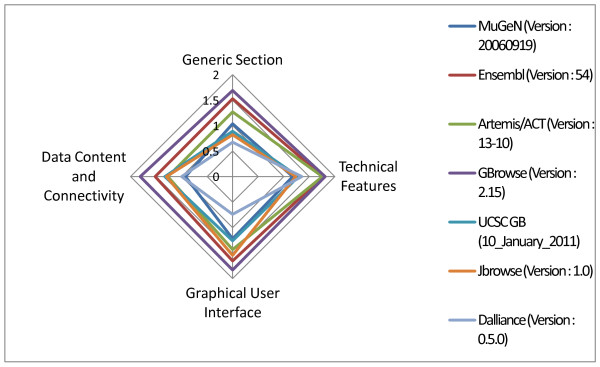
**Overall comparison of seven GBs**. The radar plot summarizes the scoring of our evaluation without any weighting of criteria. Detailed information about the evaluation presented in this figure: MuGeN (Version: 20060919 released on 2006-11-13; Evaluation Date: 2010-02-07; Evaluators: Valentin Loux, Thomas Lacroix), Ensembl (Version: 54 released on 2009-05-01; Evaluation Date: 2010-06-09; Evaluator: Thomas Lacroix), Artemis/ACT (Version: 13-10 released on 2011-01-26; Evaluation Date: 2011-02-18; Evaluator: Thomas Lacroix), GBrowse (Version: 2.15 released on 2010-09-14; Evaluation Date: 2011-02-10; Evaluator: Guérin Cyprien), UCSC genome browser (Version: 10_January_2011 released on 2011-01-10; Evaluation Date: 2011-02-23; Evaluator: Thomas Lacroix), Jbrowse (Version: 1.0 released on 2010-05-01; Evaluation Date: 2010-06-22; Evaluator: Helene Chiapello), Dalliance (Version: 0.5.0 released on 2010-09-11; Evaluation Date: 2011-02-11; Evaluator: Valentin Loux).

The evaluations have been carried out between February 2010 and February 2011. Dates of evaluations and names of evaluators are indicated at http://genome.jouy.inra.fr/CompaGB and in the legend of the Figure 1. The evaluations have been performed by a single evaluator in each case, with the exception of MuGeN (two evaluators). The evaluations were systematically sent to the team that developed the software for validation.

Figure 1 summarizes the scoring (without weighting of the criteria) for the seven compared genome browsers. Complete results by category of criteria, or user profiles as well as customized comparisons are available on the public web site. Here, only a few general features of the seven compared GB are discussed. In a nutshell, we observe that Gbrowse or Ensembl offer the best results with respect to the evaluated criteria. For instance, they both offer the possibility to be locally installed, thus ensuring privacy, and/or to be installed as a public web site. They both support the DAS (Distributed Annotation System) protocol, a clear advantage for integrating annotation spread over different locations. Concerning ergonomics and ease of utilization (criteria of the GUI section, typically important for a biologist), we observe again that Ensembl and Gbrowse offer the most user-friendly interface associated with a good user support, the ability to perform different searches and many export facilities.

JBrowse and Dalliance offer a smooth and animated panning, zooming, navigation, and track selection. They were originally built to make the most out of Ajax technologies and therefore provide a modern web interface with a high level of interactivity. Both projects are recent compared to GBrowse or Ensembl so their functionalities are not as complete. Dalliance is a DAS client that relies on this standard for displaying the data. MuGeN is a great tool to display GenBank or additional annotations on microbial genomes, buts its navigation features are limited and it is not designed to load higher eukaryote genomes. The UCSC browser natively displays a broad range of human annotations, including cross-species comparisons. UCSC browser's underlying strategy focuses upon centralizing data on UCSC servers and, as far as we know, no external lab has installed it locally for the purpose of storing and browsing their own data. Artemis/ACT is the only choice among our selection to allow the annotation of genome sequences. The latest release of Artemis/ACT introduces a view to display alignments of reads produced by Next Generation Sequencers onto a reference genome. Beyond these general results, we believe that each GB has strengths and weaknesses depending on the context and purpose it is used for. For this reason, we encourage users of GBs to perform their own customized comparison using the CompaGB framework.

## Discussion

The aim of this work is two-fold: (i) to improve the quality, richness and reliability of GB evaluations and (ii) to promote software reusability and qualification procedures in the field of genomics. This does not mean that the used methodology makes GB evaluations unbiased since evaluations reflect to a large extent the perception and the background of the evaluators. For this reason, we encourage the community to post comments and/or suggestions of modifications and/or supplementary evaluations of the current evaluated GB on the CompaGB web site according to their own experience.

Presenting an exhaustive panorama of the recent GB releases with a broad representation of the views from different user communities is a huge task, which is not within the scope of this paper. We are aware that a unique local team is not enough to meet three major requirements: (1) evaluating exhaustively GB softwares, (2) maintaining evaluations up to date with software's releases and (3) providing a broad representation of the views from the different user communities. Our answer to this was to make CompaGB an open framework in which anyone interested could participate. We welcome any contribution to CompaGB. We wrote a guideline to help new evaluators.

At this date, the evaluation process we developed is very complete and detailed. For this reason it might appear time-consuming to some participants though it is very dependant of the evaluator background and the GB complexity. We are aware of this weakness and we are planning to propose a simplified evaluation process in the near future.

## Availability and requirements

• **Project name**: CompaGB

• **Project home page**: http://genome.jouy.inra.fr/CompaGB

• **Operating system(s)**: Platform independent

• **Programming langage**: Java

• **Other requirements**: supported web browsers: Firefox3+, chrome, safari3+, Internet Explorer8+

• **License**: Affero GPL

• **Any restrictions to use by non-academics**: none

## List of abbreviations used

GBs: genome browsers

## Competing interests

The authors declare that they have no competing interests.

## Authors' contributions

TL, VL, HC, JFG and AG participated in the design of the QSOS criteria and in the results analysis and interpretation. VL performed some GBs evaluations. TL performed GB evaluations, developed the website and drafted the manuscript. HC initiated the study, performed some GBs evaluations and drafted the manuscript. All authors read and approved the final manuscript.
